# Supplementary material for: Content-rich biological network constructed by mining PubMed abstracts
Source: BMC Bioinformatics. 2004 Oct 8;5:147. doi: 10.1186/1471-2105-5-147 (PMC528731; doi:10.1186/1471-2105-5-147)
Supplement: Additional File 2 — The original results of the above study (non-essential files are deleted to keep the file size under the limit set by BMC bioinformatics). [file 1471-2105-5-147-S2.bz2 › chilibotAdditionalFile2/dip05/646ID8430315E11033/html/CRR_GLPK.html]

 


 **CRR** and **GLPK** 
  
Found 1 abstracts in PubMed, retrieved 1.  
 

 What does Google say? 
 PDF only 
| .edu only 

---

- Mol Gen Genet, 1995   **Regulation of glycerol and maltose uptake by the IIAGlc like domain of IINag of the phosphotransferase system in Salmonella typhimurium LT2.** .
  In Enterobacteriaceae the nonphosphorylated form of IIAGlc of the phosphoenolpyruvate dependent phosphotransferase system PTS can inhibit the uptake and subsequent metabolism of glycerol and maltose by binding to, and inhibiting, glycerol kinase **[GLPK]** and the MalK protein of the maltose transport system, respectively.
  In this report we show that the IIAGlc like domain of the membrane bound IIN acetylglucosamine IINag of the PTS can replace IIAGlc in a Salmonella typhimurium **crr** mutant strain that lacks all soluble IIAGlc.
  The inhibition was most severe in cells which were partially induced for the glycerol or maltose uptake systems.
  The Streptococcus thermophilus lactose transporter LacS, which also contains a IIAGlc like domain, could not replace IIAGlc.
  Neither IINag nor LacS could replace IIAGlc in activation of adenylate cyclase.
